# Assessment of asymptomatic *Plasmodium* spp. infection by detection of parasite DNA in residents of an extra-Amazonian region of Brazil

**DOI:** 10.1186/s12936-018-2263-z

**Published:** 2018-03-14

**Authors:** Filomena E. C. de Alencar, Rosely dos Santos Malafronte, Crispim Cerutti Junior, Lícia Natal Fernandes, Julyana Cerqueira Buery, Blima Fux, Helder Ricas Rezende, Ana Maria Ribeiro de Castro Duarte, Antonio Ralph Medeiros-Sousa, Angelica Espinosa Miranda

**Affiliations:** 1 Graduate Programme in Infectious Diseases, Federal University of Espírito Santo, Vitória, Brazil; 2 Protozoology Laboratory, Institute of Tropical Medicine, University of São Paulo, São Paulo, Brazil; 3 Entomology and Malacology Unit, Espírito Santo State Department of Health (SESA), Vitória, Brazil; 4 Superintendency for the Control of Endemic Diseases (SUCEN), São Paulo State Department of Health, São Paulo, Brazil; 5 Faculty of Public Health, University of São Paulo, São Paulo, Brazil

**Keywords:** Malaria, *Plasmodium vivax*, *Plasmodium malariae*, Asymptomatic carrier, PCR

## Abstract

**Background:**

The hypotheses put forward to explain the malaria transmission cycle in extra-Amazonian Brazil, an area of very low malaria incidence, are based on either a zoonotic scenario involving simian malaria, or a scenario in which asymptomatic carriers play an important role.

**Objectives:**

To determine the incidence of asymptomatic infection by detecting *Plasmodium* spp. DNA and its role in residual malaria transmission in a non-Amazonian region of Brazil.

**Methods:**

Upon the report of the first malaria case in 2010 in the Atlantic Forest region of the state of Espírito Santo, inhabitants within a 2 km radius were invited to participate in a follow-up study. After providing signed informed consent forms, inhabitants filled out a questionnaire and gave blood samples for PCR, and thick and thin smears. Follow-up visits were performed every 3 months over a 21 month period, when new samples were collected and information was updated.

**Results:**

Ninety-two individuals were initially included for follow-up. At the first collection, all of them were clearly asymptomatic. One individual was positive for *Plasmodium vivax*, one for *Plasmodium malariae* and one for both *P. vivax* and *P. malariae*, corresponding to a prevalence of 3.4% (2.3% for each species). During follow-up, four new PCR-positive cases (two for each species) were recorded, corresponding to an incidence of 2.5 infections per 100 person-years or 1.25 infections per 100 person-years for each species. A mathematical transmission model was applied, using a low frequency of human carriers and the vector density in the region, and calculated based on previous studies in the same locality whose results were subjected to a linear regression. This analysis suggests that the transmission chain is unlikely to be based solely on human carriers, regardless of whether they are symptomatic or not.

**Conclusion:**

The low incidence of cases and the low frequency of asymptomatic malaria carriers investigated make it unlikely that the transmission chain in the region is based solely on human hosts, as cases are isolated one from another by hundreds of kilometers and frequently by long periods of time, reinforcing instead the hypothesis of zoonotic transmission.

**Electronic supplementary material:**

The online version of this article (10.1186/s12936-018-2263-z) contains supplementary material, which is available to authorized users.

## Background

With an estimated 212 million new cases in 2015, leading to 429,000 deaths, malaria is an infectious disease which has an enormous global economic and social impact [1]. Efforts to control the disease over the years have resulted in a significant reduction in its incidence and specific mortality. However, these achievements are threatened not only by the development of resistance to drugs and insecticides of the protozoans [1, 2] and mosquito vectors [3, 4] involved in the transmission chain, but also by the limited financial resources available to implement the required measures [1, 5].

Malaria control strategies target the various components of the transmission chain: the protozoans of the genus *Plasmodium*, the anopheline mosquito vectors, and the susceptible or infected humans. However, this transmission chain has several complexities that can compromise the effectiveness of control measures. With the development of molecular biology techniques, the number of *Plasmodium* species known to be able to cause infections in humans has increased from four in the oldest references, to six in the most recent, with the recent split of *Plasmodium ovale* into two different species [6–9]. One of these new species, *Plasmodium knowlesi*, was originally identified as the species that caused simian malaria in Southeast Asia, but is now known to also play an important role in the disease in humans living in the same region [10–12]. Like *P. knowlesi*, other species may act as parasites in humans and non-human primates, forming a zoonotic transmission chain. This makes it necessary to develop more complex measures to either eliminate the disease or to at least impede its spread, as the elimination of diseases with a zoonotic cycle might only be attainable at an unacceptably high cost to the environment.

Another factor that makes the malaria transmission chain more complex is the role played by asymptomatic carriers. These are known to exist ubiquitously, and their frequency varies according to the degree of endemicity [13–32]. They very often present with subpatent parasitaemia, which can only be detected by molecular methods, making identification of the infection difficult, and interfering with efforts to eliminate the disease [15, 21, 23, 29, 30].

While more than 99% of malaria cases in Brazil are restricted to the Amazon region [32], residual cases have been reported in areas of the Atlantic Forest in various states in other regions [15–17, 20, 25, 32], where the characteristics of the transmission cycle are different. For example, outbreaks do not generally occur, the incidence and parasitaemia are very low, the clinical symptoms are mild, and the species responsible for infection are *Plasmodium vivax* and *Plasmodium malariae* [17]. However, the most distinctive characteristic of malaria in this particular region is the vector. Namely, the anophelines involved in transmission of the disease belong to the subgenus *Kerteszia*, which breeds in the axils of bromeliads, and therefore has a very close relationship with the Atlantic Forest ecosystem [20, 33–37]. Simian malaria also occurs in these regions and is caused by parasites that are now known to belong to the same species that cause disease in humans, and to be transmitted by the same vectors [33, 35, 38–47]. While these characteristics would lend strong support to the hypothesis of zoonotic transmission, more conclusive evidence is required to corroborate it. Consideration should also be given to the alternative hypothesis, i.e. that the low-incidence malaria is being maintained by a contingent of unidentified asymptomatic carriers.

A previous study in the malaria transmission area in the state of Espírito Santo detected *Plasmodium* spp. DNA in the blood samples of 48 out of 1527 asymptomatic inhabitants of the region, corresponding to a prevalence of 3.1% [17]. In the present study, a cohort from an area in this endemic region was followed up longitudinally to confirm the previously established prevalence and determine the incidence and persistence of the carrier state. Previously collected data on vector density, the demographics of the region, and the transmission parameters available from the literature were used to calculate the basic reproductive rate for new infections and thus determine whether the transmission chain in the region could be sustained by human hosts and vectors alone. To distinguish between the two hypotheses, the natural human recovery rate without treatment, as well as a scenario in which all human *Plasmodium* carriers are asymptomatic were considered.

## Methods

### Study area

**Fig. 1:**
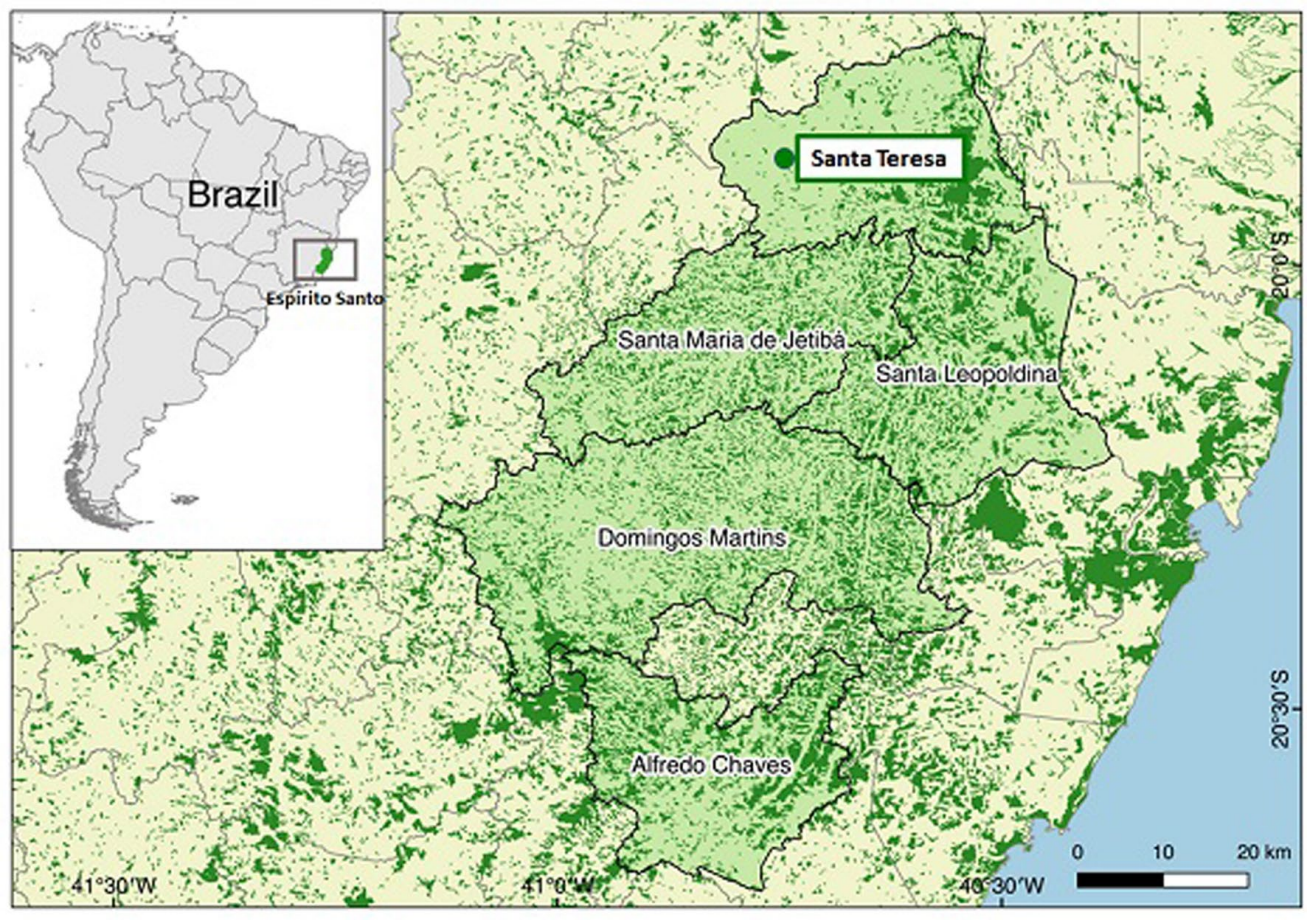
Map showing the study area in Espírito Santo, Brazil

The state of Espírito Santo is the Brazilian state with the most cases of residual malaria in the Atlantic Forest [17]. The disease occurs in the mountainous region of the state, which lies no more than 50 km on average from the coast. Between 17 and 68 cases were recorded every year between 2007 and 2015 [48] in an area covering approximately 5343 square km. The highest frequency of cases was recorded in 2008. After that point, the incidence decreased, and has since remained stable. During the study period, 32, 31, and 17 cases were recorded in 2010, 2011, and 2012, respectively. After the study, the figures were 35, 30, and 48 in 2013, 2014, and 2015, respectively [48]. A survey carried out in this area between 2001 and 2004 [17] identified *P. vivax* in 48 out of 51 symptomatic individuals by both microscopic examination of blood films and polymerase chain reaction (PCR). *Plasmodium malariae* was identified by PCR in one sample, while in two samples blood smear tests revealed parasites morphologically similar to *P. vivax*, but with a negative PCR result. The cases were detected in nine municipalities located between 19.6° and 20.6° south latitude and 40.6° and 41° west longitude [17] (Fig. 1). In the study area, the landscape is irregular with mean altitude of around 800 meters. Despite the tropical climate, lower temperatures of around 15 °C are registered from May to August because of the high altitude. Human dwellings are close to the well-preserved tropical forest with a fauna consisting of birds, reptiles, and small mammals, including simians from the Cebidae and Atelidae families. In this study, 92 of the approximately 120 individuals living within a 2 km radius of the home of the first person diagnosed with malaria by the health authorities in 2010 were approached.

### Design

Quarterly assessments of inhabitants in the region where the first malaria case detected in 2010 occurred were scheduled, resulting in a total of eight assessments over 21 months. Before the first assessment the participants signed a voluntary informed-consent form. During each assessment, a questionnaire was filled out covering demographic data and any instances since the previous collection of febrile illnesses, as well as thick-smear tests for malaria and trips outside the area where the participant lived. A total of 5 mL of blood was then collected in Vacutainer tubes containing EDTA, thick and thin peripheral blood smears were prepared, and abdominal palpation for splenic enlargement was performed.

The blood collected was centrifuged at 300 g for 10 min in the Protozoology Laboratory at the Tropical Medicine Unit, Federal University of Espírito Santo, to separate the plasma from the red blood cells. The latter were then aliquoted and stored at -20 °C in Eppendorf tubes, which were sent to the Protozoology Laboratory at the São Paulo Institute of Tropical Medicine, where DNA was extracted and PCR was performed.

### Thick and thin smears

Thick and thin blood smears were prepared following the method recommended by the World Health Organization [49]. The smears were examined under an optical microscope with a 100× objective lens. The results were based on examination of at least 200 microscopic fields.

### Amplification of *Plasmodium* DNA

Genomic DNA was extracted from the samples with a NucleoSpin Tissue purification kit (Macherey–Nagel) following the manufacturer’s instructions, and amplified following Win et al. [50]. Briefly, nested-PCR, which consists of two rounds of amplification, was carried out with primers that target the *Plasmodium* 18S RNA gene subunit. The first step was carried out in a final volume of 20 μL containing 0.8 μL of each primer, P1UP and P2 (10 μM), 0.25 μL of dNTP mix (10 mM each, Thermo Fisher Scientific), 2 μL of 10 × PCR buffer, 1 μL of MgCl_2_ (50 mM), 0.16 μL of Platinum Taq DNA polymerase-Invitrogen (5 U/μL) and 5 μL of DNA. The amplification was run in an Applied Biosystems thermocycler with the following programme: 92 °C for 2 min, 35 cycles of 92 °C for 30 s and 60 °C for 90 s, and one cycle of 60 °C for 5 min.

The product from the first step was diluted at a ratio of 1:50 in sterile water and used in the second step with the P1 (genus–specific) primer and one of the three reverse species-specific primers (V1—*P. vivax*, F2—*Plasmodium falciparum* or M1—*P. malariae*).

This step was carried out in a final volume of 20 μL, containing 2 μL of each primer (10 μM), 0.5 μL of dNTP (10 mM each, Thermo Fisher Scientific), 2 μL of 10 × PCR buffer, 0.16 μL of Taq DNA polymerase (5 U/μL), and 2 μL of the diluted final product from the first step. The second amplification was carried out using the same equipment as the first, and the cycling parameters were 92 °C for 2 min, followed by 18 cycles of 92 °C for 30 s and 60 °C for 1 min, and one cycle of 60 °C for 5 min. The amplified products of the second step correspond to species–specific fragments of about 100 bp.

The primers used were:

- P1UP (F): 5′ TCC ATT AAT CAA GAA CGA AAG TTA AG 3′
- P2 (R): 5′ GAA CCC AAA GAC TTT GAT TTC TCA T 3′
- P1 (F): 5′ ACG ATC AGA TAC CGT CGT AAT CTT 3′
- V1 (R): 5′ CAA TCT AAG AAT AAA CTC CGA AGA GAA A 3′
- F2 (R): 5′ CAA TCT AAA AGT CAC CTC GAA AGA TG 3′
- M1 (R): 5′ GGA AGC TAT CTA AAA GAA ACA CTC ATA T 3′

The amplified product was run on 2% agarose gel at 80 V for 40 min. The gel was stained using ethidium bromide, and the bands were visualized under a UV transilluminator. DNA extracted from peripheral blood of *P. vivax* malaria patients treated at the São Paulo Superintendency for the Control of Endemic Diseases (SUCEN), from *P. falciparum* cultures, and from blood smears positive for *P. malariae* provided by the Centers for Disease Control and Prevention (CDC) was used as a control.

### Application of the mathematical transmission model

Based on entomological and demographic information for the study area, and transmission parameters available in the literature, the basic reproductive rate (R_0_) for the mathematical malaria transmission model proposed by Anderson and May [51] was calculated. This is a deterministic model, and R_0_ is calculated using the following equation:

$${\text{R}}_{0} = \frac{{\left( {\frac{M}{N}} \right)b^{2} T_{mh} T_{hm} }}{\gamma \mu }e^{ - \mu p} e^{ - \gamma q} ,$$

where, R_0_ = Basic reproductive rate, N = Estimated population size, M = Abundance of *Anopheles cruzii*, the vector in the region, µ = Mortality rate of *An. cruzii*, T_MH_ = Probability of transmission of *Plasmodia* from *An. cruzii* to humans, T_HM_ = Probability of transmission of *Plasmodia* from humans to *An. cruzii*, γ = Human recovery rate, *b *= Average *An. cruzii* daily biting rate, *q* = Incubation period for *P. vivax* in humans, p = Extrinsic incubation period for *P. vivax*.

The basic reproductive rate represents the number of secondary cases that will be generated from an infected individual. In theory, for deterministic transmission models, the number of infected individuals in the population is increasing when R_0_ > 1 and decreasing when R_0_ < 1. For endemic diseases, R_0_ tends toward an equilibrium value, i.e. close to 1 [51].

### Calculation of sample size

The sample size was calculated using a value of 3.1% for the frequency of PCR-positive blood samples, the value found in the survey carried out between 2001 and 2004 [17]. It was assumed a priori that the carrier state lasts only a short amount of time, and that most individuals either develop the disease or recover spontaneously [13]. As a consequence, the annual incidence must be close to the frequency established in an earlier survey [17]. Therefore, considering an incidence of 3.1% and a 95% confidence interval, a sample of 90 patients would result in an estimate per interval of 0–7% (Epi Info version 3.4.1). This sample size was calculated taking into consideration *P. vivax* as the main parasite in terms of public health importance in the region, although it might overestimate the incidence of *P. malariae*, as this parasite can persist for longer periods in the bloodstream of infected individuals.

### Data analysis

Continuous quantitative variables were expressed as medians and interquartile ranges because of the high variability of the data, and categorical variables were expressed as absolute and relative frequencies. The data were analysed by means of SPSS 17.0.

## Results

### Characteristics of the cohort

In March 2010, after the first case of malaria in the study region was reported that year, 92 individuals living within a 2 km radius of the location where the case occurred were included in the study. They were assessed at 3-month intervals on eight occasions, giving a total of 21 months of follow-up. The total number of individuals assessed was expressed in person-years and not all individuals attended all the assessments. At the initial assessment, demographic data and information about participants’ occupations and leisure activities, as well as any habits potentially connected with malaria transmission were collected (Table 1). None of the individuals’ thick or thin blood smears were positive for protozoans, and none of them presented any symptoms compatible with malaria at any of the assessments. Abdominal palpation for splenic enlargement was performed at every assessment, but failed to reveal any cases of enlarged spleen.

**Table 1:** Characteristics of the cohort of 92 individuals living in an area of the Atlantic Forest with residual malaria who were assessed at 3-month intervals between March 2010 and December 2011

Characteristic	Median (IQR)	Frequency	Percentage
Age (years)	32 (14.2–54.7)	–	–
Gender			
Male	–	49	53.3
Female	–	43	46.7
Place of birth			
Same municipality	–	47	51.1
Other state	–	20	21.7
Other municipality without malaria	–	16	17.4
Other municipality with malaria	–	8	8.7
Other country	–	1	1.1
Occupations classified in terms of their relationship with rural areas			
Agricultural	–	51	55.4
Non-agricultural and unrelated	–	28	30.4
Non-agricultural but related	–	13	14.1
Incursion into the forest in the previous 2 months			
Yes	–	43	46.7
No	–	49	53.3

*IQR* Interquartile range

The most common occupations were farmer (43 individuals, or 46.8%), student (22 individuals, or 23.9%), and housewife (10 individuals, or 10.9%). The remaining occupations were self-employed (two), cook, maid (two), transportation supervisor, plasterer, timber worker, minor (three), member of the armed forces, driver, machine operator, agricultural producer, teacher, and electronics technician.

Thirty-two individuals had lived in the area where the assessments were carried out for less than 5 years. Of these, eleven came from other areas in the same municipality, nine from malaria-free municipalities in the Atlantic Forest, five from other states, and two from other municipalities with malaria in the Atlantic Forest. No information was available for the remaining five. None of those who said they had come from other states were from the Amazon region, and none of the individuals had travelled to the Amazon region during or before the study period.

Three individuals reported having had malaria before, one in 2002 and two in 2004. The diagnosis was based on the result of a thick blood smear test, and the parasites were morphologically consistent with *P. vivax*.

Thirteen individuals reported having had fever episodes in the previous 2 years. Of these, two said that the fever had lasted more than 3 days (15 and 7 days, respectively). The patient who reported a 7-day fever was the only individual to report having had a fever on two separate occasions, the second lasting 3 days. None of these individuals were investigated for malaria by the local health services when they had fever.

Ten individuals reported having had fever episodes between the first and second assessments; of these, three said that the fever had lasted more than 4 days (6, 7 and 14 days). A thick blood smear for the individual who had had a fever for 14 days was negative when examined by local health staff. The number of individuals who reported having had a fever between subsequent assessments was two, three, four, six, three, and zero for each of the assessments, respectively. Malaria was not diagnosed at any of the assessments, and the febrile episodes improved spontaneously. Three individuals reported prolonged febrile episodes, one between the fourth and fifth assessments, lasting 21 days, one between the fifth and sixth assessments, lasting 15 days, and one between the sixth and seventh assessments, lasting 30 days.

### Prevalence and incidence of asymptomatic *Plasmodium* DNA carrier state

At the first assessment, one individual was positive for *P. vivax*, one for *P. malariae* and one for *P. vivax* and *P. malariae*, corresponding to a prevalence of 3.4% in the population sample (2.3% for each species). During follow-up, four new PCR-positive cases (two for *P. vivax* and two for *P. malariae*) were recorded, corresponding to an incidence of 2.5 infections per 100 person-years or 1.25 infections per 100 person-years for each species in the population sample analysed (Table 2) (Additional file 1).

**Table 2:** Results of the 3-monthly assessments between March 2010 and December 2011 for the PCR-positive individuals among 92 study participants living in an area of the Atlantic Forest with residual malaria

Individual (no.)	Assessment							
	1	2	3	4	5	6	7	8
33	PM/PV	PM	–	PM	–	PM	PM	PM
38	–	–	–	PV	A	–	–	–
56	–	PV	–	PV	–	–	–	–
69	PM	–	–	–	–	–	–	–
71	PV	–	–	–	PM	–	PM	–
84	–	PM	PM	–	–	PM	–	–

PM *P. malariae*, PV *P. vivax*, − negative result, A absent

### Application of the mathematical transmission model

**Fig. 2:**
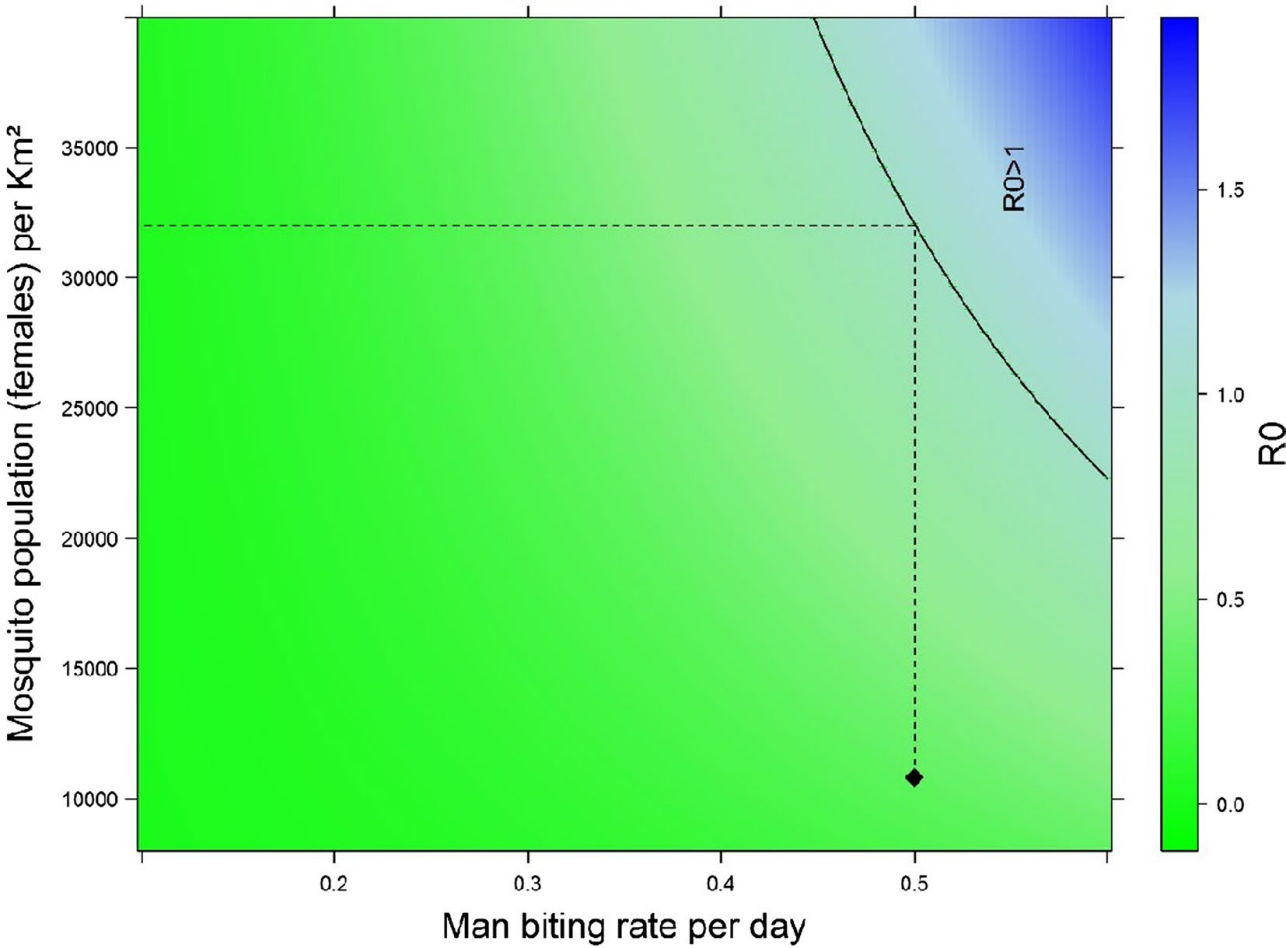
Schematic representation of the basic reproductive rate (R_0_) for the study region estimated using the mathematical model proposed by Anderson and May [51] (filled diamond). Only factors related to the vector are taken into account. The solid line represents the threshold for R_0_ = 1. The dashed line indicates how much the vector population should increase to reach this threshold

R_0_ was calculated using the transmission parameters for *P. vivax*, because it is the *Plasmodium* species most frequently associated with symptomatic cases in the study region (Table 3 and Fig. 2). These parameters yielded a value of R_0_ = 0.337, suggesting that, given the estimated vector density, human hosts alone would not be sufficient to maintain endemic malaria in this region.

**Table 3:** Parameters used to calculate R_0_ for the mathematical model

Parameter	Estimate	Basis for the estimate
N	15 inhabitants/km^2^	Value obtained by dividing the rural population in 2010 by the area of the municipality based on Brazilian Institute of Geography and Statistics (IBGE) figures [52]
M	10,815 specimens/km^2^	Value obtained from entomological studies carried out in the area [36, 37] and by applying linear regression following Zippin [53]
μ	0.68	Bona and Navarro-Silva [54]
T_MH_	0.022	Chitnis et al. [55]; Nedelman [56]
T_HM_	0.24	Chitnis et al. [55]; Nedelman [56]
γ	0.0055 (180 days)	Value obtained from the observed frequency of asymptomatic individuals and the results reported by Chitnis et al. [55]
*b*	0.5	Santos [57]; Laporta et al. [58]
*q*	10 days	Chitnis et al. 2008 [55]
p	8 days	Santos [57]; Laporta et al. [58]

*N* Estimated population size, *M* Abundance of *An. cruzii*, *µ* Mortality rate of *A. cruzii*, *T*_*MH*_ Probability of transmission of *Plasmodium* from *An. cruzii* to humans, *T*_*HM*_ Probability of transmission of *Plasmodium* from humans to *A. cruzii*, *γ* Human recovery rate, *b* Average *An. cruzii* daily biting rate, *q* Incubation period for *P. vivax* in humans, *p*  Extrinsic incubation period for *P. vivax*

## Discussion

In this follow-up of a cohort of individuals living in an extra-Amazonian area of Brazil endemic for malaria, the initial prevalence of asymptomatic *Plasmodium* carriers was 3.4%. The incidence in the population sample studied was 2.5 infections per 100 person-years, or 1.25 infections per 100 person-years for each species (*P. vivax* and *P. malariae*). A prevalence of this magnitude is similar to that reported by Cerutti et al. [17] in 1527 asymptomatic individuals investigated in an earlier study in the same region of the Atlantic Forest in the state of Espírito Santo. Although the data are for a small population sample, the present study is noteworthy because it is the first to document the incidence of asymptomatic carriers in a longitudinal follow-up outside the Amazon region. In contrast, other studies of infected individuals in different areas of Brazil outside the Amazon region are based on cross-sectional or secondary data, and therefore do not allow the incidence to be calculated [15–17, 20, 25, 59–65].

Throughout the follow-up, only three asymptomatic *Plasmodium* carriers remained positive in more than three blood collections, and all those positive for *Plasmodium* turned negative spontaneously, with the exception of a single carrier who initially tested positive for *P. vivax/P. malariae*, but in subsequent collections only tested positive for *P. malariae*. All four individuals who were positive only after the first collection subsequently tested negative for *Plasmodium* DNA.

Individual no. 33 remained positive for *P. malariae* DNA between the first collection and the end of the study period, with the exception of two collections in which the PCR was negative. The parasites that cause quartan malaria, such as *P. malariae*, appear to be the best adapted to their hosts, who can have a chronic infection for decades without symptoms [66]. These infections can go largely unnoticed as the vast majority of symptomatic cases detected are caused by *P. vivax* [17].

Three of the individuals enrolled in the study reported having had malaria more than 5 years previously, when it was diagnosed based on the result of a thick blood smear that showed parasites morphologically consistent with *P. vivax*. One of these, individual no. 84, who reported a previous episode of malaria in 2004 and was treated in accordance with Ministry of Health recommendations, was negative in the first assessment in this study, and positive for *P. malariae* DNA in subsequent assessments.

Although not probable, it is possible that the infection could have persisted in individual no. 84, producing gametocytes that could help to maintain the transmission cycle. Alves et al. [67] cited unpublished observations that asymptomatic individuals could remain infective, though with less capacity to infect mosquitoes than symptomatic ones. However, their limited capacity of infection would be compensated by the long periods of the shedding of gametocytes. Even considering such a possibility, the mathematical model applied in this study predicted a period of asymptomatic infection of 6 months, greater than the 2 months period observed by Alves et al., after which 40% of the asymptomatic individuals became negative [67]. In other words, had individual no. 84 not cleared his parasitaemia after treatment, staying infected by *P. vivax*, the probability of remaining infected would have decreased as time passed, along with his capacity of remaining infective. Even if his infection had persisted for 6 months, the mathematical model would have still been applicable and the prediction would have remained valid.

There is also the possibility that individual no. 84 was in fact infected by *P. malariae* instead of *P. vivax* when diagnosed by the local health system in the first place. As infections by *P. malariae* can persist much longer than those by *P. vivax* [66], the DNA amplified in the present study could have been the same as the parasite detected in his previous infection. However, as demonstrated by Cerutti et al. [17], infections by *P. malariae* are much less frequent than those by *P. vivax* in the Atlantic Forest system of Espírito Santo, being less important from an epidemiological point of view. Even such an unlikely scenario would not have interfered with the mathematical prediction of the present study, which, incidentally, was constructed taking *P. vivax* into consideration.

Of the thirteen individuals who presented with fever during the study period, none had splenomegaly, and only one individual who had a prolonged episode of fever was tested using a blood smear when he was seen by a local physician, with a negative result. In all the followed-up patients who had fever at some time, the condition resolved itself spontaneously without the use of antimalarial medication.

Since none of the individuals migrated to areas outside the Atlantic Forest, particularly the Amazon region, at any time before or during the study, it is clear, despite the small number of individuals with samples positive for *Plasmodium* DNA and the small sample size, that the study area is indeed endemic for malaria, albeit at a very low level.

In light of the value of R_0_ (0.337) and the fact that, in theory, R_0_ < 1 indicates that the number of infected individuals is decreasing in the study population, it is reasonable to suggest that the persistence of endemic malaria in the study area cannot be explained only by the presence of asymptomatic infected individuals but would require an additional reservoir, such as non-human primates. This explanation would currently appear to be plausible as a monkey found in the wild in the same region was positive for *P. vivax* DNA [46].

In a study by Rezende et al. [36] using PCR, *P. vivax* DNA was amplified in samples of thoraces of anopheline specimens of the subgenus *Nyssorhynchus* (*Anopheles parvus* and *Anopheles galvaoi*) from the study area, raising the possibility that this subgenus might be involved in malaria transmission in the Atlantic Forest. However, the authors conclude that the low endemicity of malaria is in itself evidence that these species do not participate in the transmission of the disease, as the low parasitaemia would make infection of anophelines with limited vector capacity and competence improbable. The absence of more competent vectors, such as *Anopheles darlingi*, in the study area leads to the conclusion that if the subgenus *Nyssorhynchus* is involved in the transmission cycle, its role is a secondary one, i.e. that of an incidental vector. Since it is possible that monkeys could be acting as reservoirs, a mathematical model that can take this into account is required. Development of an accurate model would in turn require further studies of the monkeys in the region.

*Plasmodium knowlesi* malaria has been shown to be a zoonosis. Initially believed to cause only simian malaria, *P. knowlesi* has been responsible for continued endemic human malaria in regions where there has been a clear decrease in infections by *P. falciparum* and *P. vivax*, and has come to represent an obstacle to malaria control efforts in regions of Southeast Asia, particularly Malaysia [11]. In this scenario, the arrival of humans in simian habitats and areas where plasmodia are transmitted can modify the normal transmission cycle, leading monkeys to congregate in residual patches of forest close to humans and to spend more time on the ground. Furthermore, they may change their behavior in their microhabitat, seeking out human settlements and attacking crops or food supplies near human dwellings [12]. Deane (1992) noted that infection by *Plasmodium simium* can occur in humans in Brazil, although only incidentally, and that *An. cruzii* is abundant in the Atlantic Forest region of the state of Espírito Santo [35]. The distribution of anophelines collected in the region in the entomological survey by Rezende et al. allowed the authors to infer that if *An. cruzii* is considered the probable vector in this region and prefers to remain in the tree canopy feeding on non-human primates, malaria is probably being transmitted as a zoonosis [36, 37]. The possibility that monkeys are acting as *Plasmodium* reservoirs in areas with residual malaria was also suggested by Duarte et al. in a study in which they investigated the prevalence of antibodies against circumsporozoite protein and asexual forms of *P. vivax*, *P. malariae* and *P. falciparum* in monkeys in the Atlantic Forest in the state of São Paulo [68]. Their findings suggested that monkeys in the region had contact with sporozoites from infected anophelines and developed infection. Additional evidences based on the mitochondrial genome of the parasites had recently been presented by the publications of Brasil et al. and Buery et al. [45, 46]. Kirchgatter et al., investigating the feeding preferences of *An. cruzii* females in Juquitiba in the state of São Paulo, argued against transmission by zoonosis. Specifically, they found only human blood in 26 engorged female mosquitoes and failed to find blood from monkeys, other mammals or animals of any other species in any of the specimens [69]. The mosquitoes were collected from a peridomestic environment because the chosen area consisted of rural human settlements that had undergone anthropogenic changes, and were located at various distances from residual forest patches where monkeys are often seen. However, given that the *An. cruzii* specimens were collected only from the peridomestic environment, rather than also from the forest environment, the habit of mosquitoes to feed on the closest host has to be taken into account. Collections at the forest edge, the true habitat of the monkeys, might have shown that *An. cruzii* also feeds on these non-human primates. In a recent study in a Yanomami community in the Venezuelan Amazon, 12 of 33 samples PCR-positive for *P. malariae* had 18S gene sequences identical to those in a *Plasmodium brasilianum* strain isolated from an infected monkey in French Guiana. As *P. brasilianum* is morphologically similar and, apart from a handful of mutations, almost genetically identical to *P. malariae*, the authors speculate that they are the same species. They note the ease with which plasmodia can be exchanged between humans and monkeys, and warn that there is a lack of host specificity for quartan malaria caused by *P. brasilianum*, which they consider a true zoonosis [70]. Similarly, there is also evidence to support the hypothesis that *P. simium* is a variant of *P. vivax*, with minimal differences in a few molecular markers [45, 46, 71]. Although *P. vivax* and *P. simium*, as well as *P. malariae* and *P. brasilianum*, are known to be derived from each other, questions remain as to the direction in which the transfer took place, whether from humans to monkeys or vice versa [42].

The main limitation of the present study are the characteristics of the sample, which, although large enough to allow new cases to be identified, was too small to allow the detection of possible factors associated with the risk of getting infected. As the sampling of the participants was a random process, despite being triggered by the occurrence of a symptomatic case, some infections could have gone unnoticed. However, the similarity between the prevalence found in this study and that evidenced by a much larger sample in the study of Cerutti et al. [17] indicates that the frequencies of positive results detected here are indicative of the magnitude of parasitic infections at a population level. On the other hand, parasite densities below the limit of detection by semi-nested PCR could have been missed. The question of infection detectability, particularly in asymptomatic human patients who commonly exhibit low parasitaemia, is crucial for the study of the transmission chain. Nevertheless, for *P. vivax*, the technique used is able to detect as few as 0.12 parasites/µl [72], ensuring a good level of confidence in the conclusions of this study. The application of a modern molecular tool as a qPCR could have further enhanced the detection of infections, but, unfortunately, such resource was not available at the time.

To calculate the incidence, individuals who were negative for *Plasmodium* DNA at the first collection and positive at one or more subsequent collections were counted only once. Furthermore, the infection was considered to be the same when an individual had a sample that was positive for the same species of *Plasmodium* in a subsequent assessment. In these circumstances, the fact that genotyping was not performed could be considered a limitation, as the possibility of a new infection cannot be eliminated.

In spite of these limitations, the results presented here clearly indicate a need to improve our understanding of the transmission cycle in these low-endemicity areas. Specifically, genotypic studies with both human and simian hosts, as well as human and simian vectors have to be undertaken in order to fully elucidate the role each plays in the transmission cycle. Of particular note here is the finding based on our mathematical model, namely that the local transmission cycle cannot be maintained by human-mosquito-human interactions alone.

## Conclusions

The present study detected a low frequency of asymptomatic carriers of *Plasmodium* spp. in an area endemic for malaria in the Atlantic Forest in Brazil. These results complement previous observations of a low frequency of symptomatic infections [17], and low density of anopheline vectors [36, 37]. The application of a mathematical model showed that, given these characteristics, it is improbable that the malaria cycle observed in this region could be maintained by human-mosquito-human interactions alone.

## Additional file

**Additional file 1.** Additional material.
