# Supplementary material for: Assessment of asymptomatic Plasmodium spp. infection by detection of parasite DNA in residents of an extra-Amazonian region of Brazil
Source: Malar J. 2018 Mar 14;17:113. doi: 10.1186/s12936-018-2263-z (PMC5853114; doi:10.1186/s12936-018-2263-z)
Supplement: Supplementary file 1 — Additional file 1. Additional material. [file 12936_2018_2263_MOESM1_ESM.docx]

Additional material

Nested PCR

Nested PCR was performed according to Win et al. (2002).

In the first step, a 130bp fragment of the 18S rRNA gene of *Plasmodium* was amplified. The reaction consisted of 5µl of DNA, 1x Taq reaction buffer, 2,5mM of MgCl_2_, 0.25 mM of dNTP mix (Fermentas), 0.4 µM of each primer (P1UP: 5’ TCC ATT AAT CAA GAA CGA AAG TTA AG 3’ and P2: 5’ GAA CCC AAA GAC TTT GAT TTC TCA T 3’), 1 U of Platinum ® Taq DNA Polymerase (Invitrogen) and ultraPure^TM^Water (Invitrogen) to a final volume of 20µl. Amplification was performed using the Mastercycler gradient thermocycler (Eppendorf®) with the following conditions: 92°C for 2 min, 35 cycles at 92°C for 30 s, 60°C for 90s and a final step of 60°C for 5 min.

In the second step, reactions to detect a 100bp fragment of the three *Plasmodium* species were performed separately using a genus specific forward primer (P1: 5’ ACG ATC AGA TAC CGT CGT AAT CTT 3’) and one of the specie specific reverse primers that targets *P. vivax* (V1: 5’ CAA TCT AAG AAT AAA CTC CGA AGA GAA A 3’), *P. falciparum* (F2: 5’ CAA TCT AAA AGT CAC CTC GAA AGA TG 3’ and *P. malariae* (M1: 5’ GGA AGC TAT CTA AAA GAA ACA CTC ATA T 3’). The reaction consisted of diluted amplicons (1:50) from the first step, 1xTaq reaction buffer, 2,5 mM of MgCl_2_, 0.25 mM of dNTP mix (Fermentas), 1µM of each primer, 1 U of Platinum®Taq DNA Polymerase (Invitrogen) and UltraPure^TM^Water (Invitrogen) to a final volume of 20µl. Amplification was performed using the Mastercycler gradient thermocycler (Eppendorf®) with the following conditions: 92◦C for2 min, 18 cycles at 92°C for 30 s, 60°C for 60s and a final step at 60°C for 5min.

Positive controls consisted of DNA extracted from peripheral blood of *P. vivax* malaria patients, *P.* *falciparum* cultures and a blood smear from a *P. malariae* infected monkey. Water was used as a negative control.

Nested PCR products were resolved by electrophoresis using a 2.5% agarose gel prepared with 1x tris borate EDTA buffer(TBE) and stained with ethidium bromide. The 50 bp DNA Ladder (Invitrogen) was included as a size marker. The products were visualized by UV transillumination, and the images recorded with a gel documentation system (AlphaImager®). The following image shows an agarose gel containing a *P. malariae* positive sample from this study.


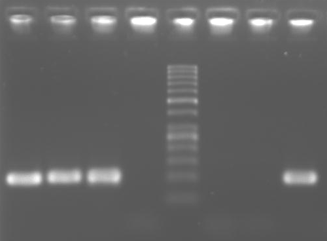


1 2 3 4 5 6 7 8

Figure. Agarose gel electrophoresis of the 100bp products from the nested PCR that targets the 18S rRNA of *P. falciparum*, *P. vivax* and *P. malariae*. Lane 1: *P. falciparum* positive control, Lane 2: *P. vivax* positive control, Lane 3: *P. malariae* positive control, Lane 4: negative control, Lane 5: molecular weight marker (50 bp DNA Ladder, Invitrogen), Lane 6 and 7: negative samples, Lane 8: *P. malariae* positive sample.
